# Extended battery longevity with external lithium supply

**DOI:** 10.1093/nsr/nwaf163

**Published:** 2025-04-25

**Authors:** Hongyan Li, Bao-Lian Su

**Affiliations:** College of Materials and Chemistry, China Jiliang University, China; Laboratory of Inorganic Materials Chemistry (CMI), Namur Institute of Structured Matter (NISM), University of Namur, Belgium; Laboratory of Inorganic Materials Chemistry (CMI), Namur Institute of Structured Matter (NISM), University of Namur, Belgium; State Key Laboratory of Advanced Technology for Materials Synthesis and Processing, Wuhan University of Technology, China

There is no doubt that it has become a fact: lithium ions will power a large part of our future. Lithium-ion (Li-ion) battery applications are becoming increasingly common, such as in the booming market of electric vehicles, home batteries and all kinds of energy-storage solutions. Li-ion batteries are pivotal in reducing global carbon emissions [1]. However, escalating demands for higher energy density, extended lifespan and sustainable material use—critical for grid storage and high-performance applications—have exposed limitations in current battery architectures [2]. Traditional Li-ion batteries rely on lithium-storing cathodes (e.g. Li–Ni–Mn–Co oxides) and lithium-free anodes (e.g. graphite) [3]. During operation, finite lithium ions shuttle between electrodes, with capacity loss occurring as lithium is consumed through irreversible side reactions [4]. This irreversible loss caps the cycle life, often rendering batteries unusable despite intact electrodes [5]. Existing prelithiation methods, which introduce lithium additives to offset losses, face challenges such as incomplete decomposition and harmful residues, failing to meet scalability and efficiency requirements [6].

**Figure 1.**
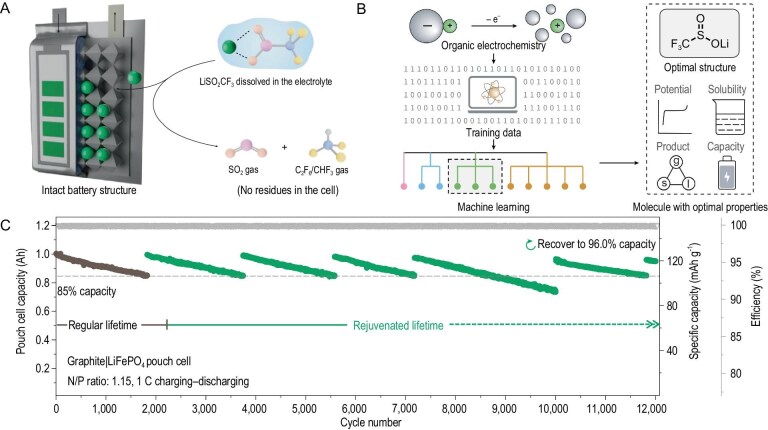
(A) External Li supply approach incorporating an organic LiSO_2_CF_3_ salt to rejuvenate cycled Li-ion batteries. (B) Discovery of the organic Li salt involved a combination of machine learning and organic electrochemistry. (C) Cycling performance of a commercial graphite|LiFePO_4_ pouch cell (1.0 Ah) with multiple Li supply. Adapted with permission from [7].

Chen *et al.* have pioneered an innovative ‘external lithium supply’ strategy, decoupling lithium replenishment from electrode materials [7]. They successfully combined artificial intelligence (AI) and organic electrochemistry to digitize molecular structures and properties. By introducing a large number of related properties into organic chemistry, electrochemistry and materials engineering technology, they built a database and used unsupervised machine learning to recommend and predict molecules. Screened from >3 million virtual molecules, they successfully obtained a never-before-reported lithium carrier molecule—lithium trifluoromethylsulfinate (LiSO₂CF₃)—called a ‘lithium drug’ (Fig. 1A and B) for the ‘precision treatment’ of used Li-ion batteries. This organic lithium salt meets many stringent requirements for external lithium supply: (i) its oxidation reaction occurs in the voltage range of 2.8–4.3 V, which is consistent with the charging window of the battery; (ii) the reaction is irreversible, preventing lithium ions from being reconsumed; (iii) the decomposition products are gaseous and can be discharged from the battery; (iv) it has good solubility in the electrolyte, facilitating diffusion and continuous decomposition; (v) it also has air stability and a high specific capacity. This organic lithium salt is dissolved directly into the electrolyte of assembled cells. During charging, the salt undergoes anodic oxidation: the (SO₂CF₃)⁻ anion decomposes into benign gases (SO₂, C₂F₆/CHF₃), releasing active lithium ions into the system. The gases are safely expelled, mimicking standard cell-formation processes, without residue and subsequent gas evolution. Crucially, this method operates at the cell level, independently of electrode chemistry, enabling lithium management as a distinct battery component.

They validated this strategy by using lithium-deficient cathode materials that were previously hindered by lithium scarcity. For instance, chromium oxide (Cr₈O₂₁) achieved a high operating voltage of 3.0 V and an exceptional energy density of 1192 Wh kg⁻¹ in an anode-less configuration, demonstrating stable cycling. Similarly, sulfurized polyacrylonitrile (SPAN)—a sustainable, low-cost material—enabled an anode-less cell with 388 Wh kg⁻¹ of energy density, retaining 80.1% capacity after 440 cycles post lithium supplementation. Notably, the approach rejuvenated a commercial 1.0-Ah graphite|LiFePO₄ cell, extending its lifespan to beyond 11 800 cycles with 96.0% capacity retention through periodic lithium replenishment (Fig. 1C), which is unachievable by simply adding additional electrolyte. This represents a 10-fold improvement over conventional cycle limits, highlighting the potential of the method to transcend existing longevity barriers.

Through their ‘precision treatment’ with a lithium carrier molecule, lithium batteries can still be close to the ‘healthy’ state when they leave the factory after tens of thousands of charge and discharge cycles, and the cycle life has been increased from the current 500–2000 cycles to >12 000–60 000 cycles. From this typeature of work, such ‘lithium drugs’ are like a ‘porter’ that only transports lithium ions and does not change the original structure and production methods of lithium batteries. In theory, these molecules will not create any new possible risks, thus achieving non-destructive battery repair. This non-invasive and rapid process preserves battery integrity without the need for disassembly. Cost analysis shows that recovery via an external lithium supply would cost just $0.9 per kilowatt-hour, while manufacturing a new battery pack would cost $132 per kilowatt-hour. This strategy could be applied in other metal-ion battery systems, such as sodium-, potassium-, zinc- and magnesium-based batteries.

By externalizing lithium supply, this breakthrough redefines battery-design paradigms. It circumvents material-specific limitations, unlocking high-performance, lithium-deficient cathodes and enabling sustainable anode-less configurations. The strategy also offers a scalable solution for revitalizing degraded commercial cells, reducing waste and enhancing resource efficiency. Furthermore, it accelerates the adoption of next-generation materials, such as transition-metal halides and organic compounds, by alleviating lithium dependency. This innovation paves the way for batteries with unprecedented lifetimes, reduced costs and improved sustainability, aligning with global decarbonization goals. Ultimately, it heralds a transformative shift in battery manufacturing, usage and recycling, with broad implications for electric mobility, renewable energy storage and portable electronics. This work also illustrates the power of the combination of AI and electrochemistry to invent more suitable battery-repair molecules, electrolytes, electrode materials and battery-management systems.
